# There Is More Than Meets the Eye: Identification of Dual Molecular Diagnosis in Patients Affected by Hearing Loss

**DOI:** 10.3390/biomedicines10010012

**Published:** 2021-12-22

**Authors:** Anna Morgan, Flavio Faletra, Giulia Severi, Martina La Bianca, Laura Licchetta, Paolo Gasparini, Claudio Graziano, Giorgia Girotto

**Affiliations:** 1 Institute for Maternal and Child Health—I.R.C.C.S. “Burlo Garofolo”, 34137 Trieste, Italy; flavio.faletra@burlo.trieste.it (F.F.); martina.labianca@burlo.trieste.it (M.L.B.); paolo.gasparini@burlo.trieste.it (P.G.); giorgia.girotto@burlo.trieste.it (G.G.); 2 U.O. Genetica Medica, IRCCS Azienda Ospedaliero—Universitaria di Bologna, 40121 Bologna, Italy; giulia_severi@aosp.bo.it (G.S.); claudio.graziano2@auslromagna.it (C.G.); 3 Reference Center for Rare and Complex Epilepsies—EpiCARE, IRCCS Istituto delle Scienze Neurologiche di Bologna, 40121 Bologna, Italy; laura.licchetta@ausl.bo.it; 4 Department of Medicine, Surgery and Health Sciences, University of Trieste, 34127 Trieste, Italy; 5 U.O. Genetica Medica, AUSL della Romagna, 47521 Cesena, Italy

**Keywords:** hereditary hearing loss, whole-exome sequencing, dual molecular diagnosis

## Abstract

Hearing loss (HL) is the most common sensory impairment, and it is characterized by a high clinical/genetic heterogeneity. Here we report the identification of dual molecular diagnoses (i.e., mutations at two loci that lead to the expression of two Mendelian conditions) in a series of families affected by non-syndromic and syndromic HL. Eighty-two patients who displayed HL as a major clinical feature have been recruited during the last year. After an accurate clinical evaluation, individuals have been analyzed through whole-exome sequencing (WES). This protocol led to the identification of seven families characterized by the presence of a dual diagnosis. In particular, based on the clinical and genetic findings, patients have been classified into two groups: (a) patients with HL and distinct phenotypes not fitting in a known syndrome due to mutations at two loci (e.g., HL in association with Marfan syndrome) and (b) patients with two genes involved in HL phenotype (e.g., *TMPRSS3* and *MYH14*). These data highlight for the first time the high prevalence of dual molecular diagnoses in HL patients and suggest that they should be considered especially for those cases that depart from the expected clinical manifestation or those characterized by a significant intra-familiar variability.

## 1. Introduction

The identification of the molecular basis of a genetic disorder is the central goal of modern medical genetics. In fact, the definition of the correct molecular diagnosis offers a series of benefits that have a tremendous impact on human life, such as understanding the pathological mechanisms, the implementation of preventive strategies, the development of personalized treatments, etc.

Recently, next-generation sequencing (NGS) technologies, and in particular whole-exome sequencing (WES), have been introduced in the diagnostic routine of Mendelian diseases, revolutionizing the approach of the study of genetic disorders. The possibility to screen multiple genes simultaneously in an unbiased method allowed the identification of the molecular cause of many rare diseases, especially those characterized by high genetic heterogeneity.

The pathologies that benefited the most from the introduction of NGS technologies are those characterized by high genetic heterogeneity and different models of inheritance such as hearing loss (HL).

HL is the most prevalent sensory impairment in both childhood and adulthood [1]. According to the World Health Organization (WHO), over 430 million people (i.e., more than 5% of the world’s population) have disabling HL, and this number is estimated to almost double by 2050 (https://www.who.int/news-room/fact-sheets/detail/deafness-and-hearing-loss, accessed on 24 November 2021). It is extremely heterogeneous both from the clinical and genetic point of view and can be classified in many categories according to different parameters, such as (1) the degree and configuration of loss, (2) the location of the damage causing the impairment, and (3) the age of onset. At least 50% of congenital or childhood HL is attributable to genetic causes, leading to hereditary hearing loss (HHL) [2].

HHL can be further classified as syndromic (SHL), when HL is accompanied by other clinical features and non-syndromic (NSHL) [3]; it is estimated that SHL accounts for ~30% of the total cases, while NSHL for the remaining ~70%. However, some reports are now calling into question these percentages, describing several cases of apparent NSHL, which should instead be classified as SHL [4,5]. As regards genetics, to date, about 123 genes (51 autosomal dominant (DFNA) genes, 77 autosomal recessive (DFNB) genes, 5 X-linked genes) have been reported as causative of NSHL (Hereditary Hearing Loss Homepage; http://hereditaryhearingloss.org/, accessed on 24 November 2021), and more than 400 syndromes associated with HL [6].

Thanks to the application of a multi-step approach, here we describe the identification of an additional peculiar scenario, the one of dual molecular diagnosis in patients affected by HHL, i.e., the presence of pathogenic variations at two distinct loci that lead to the expression of two Mendelian conditions, which segregate independently.

## 2. Materials and Methods

### 2.1. Samples Collection

During the last year, a total of 82 subjects affected by HL have been recruited at the IRCCS “Burlo Garofolo” in Trieste and at the Policlinico S.Orsola-Malpighi in Bologna (Italy).

All the participants underwent an accurate clinical evaluation. In particular, a complete medical history has been collected to exclude HL due to infections, trauma, or other non-genetic causes. All the patients underwent pure tone audiometric testing (PTA) or auditory brainstem response (ABR) (based on the probands’ age) to define the degree of HL according to the international guidelines described by Clark (1981) [7]. Neurological and ophthalmological examinations, electrocardiogram, kidney ultrasonography, brain magnetic resonance imaging (MRI) and computerized tomography (CT) scan, and thyroid function assessment were performed as well.

Based on the clinical findings, patients have been classified as likely NSHL (N = 67) or likely SHL (N = 15). Moreover, based on the inheritance pattern, familial cases were further categorized as: presumed autosomal recessive (N = 4), presumed autosomal dominant (N = 14), or sporadic cases (N = 64).

Written informed consent was obtained from all the participants; in the case of minors, their next of kin provided written informed consent. The study was conducted in accordance with the tenets of the Helsinki Declaration and was approved by the Ethics Committee of IRCCS-Burlo Garofolo of Trieste and of Policlinico S.Orsola-Malpighi of Bologna (Italy) (code 242/07, approved in 2007).

**Figure 1:**
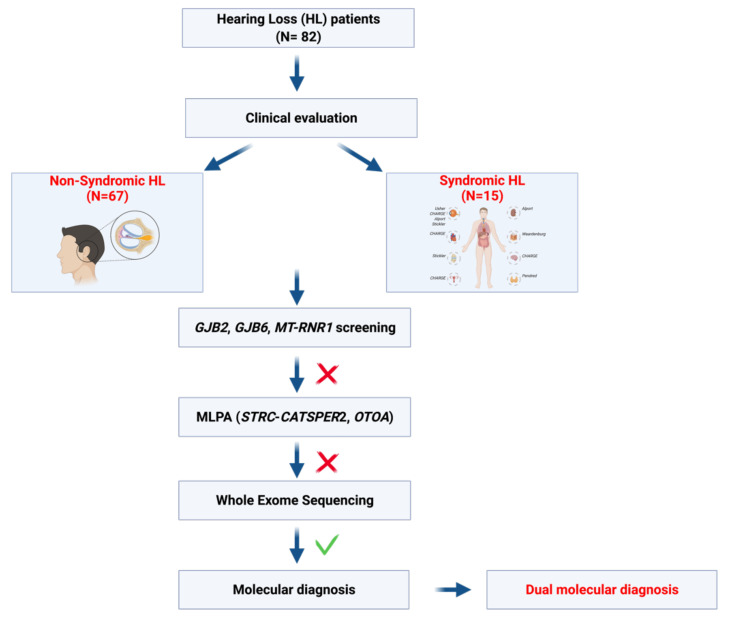
Schematic representation of the multi-step approach applied in the present study. During the last year, a total of 82 patients displaying HL have been enrolled. All of the patients underwent a careful clinical evaluation to distinguish between non-syndromic HL and syndromic HL. Afterward, individuals were screened for mutation in *GJB2*, *GJB6*, and *MT-RNR1* genes and for deletions in *STRC-CATSPER2* and *OTOA* genes. Subsequently, whole-exome sequencing has been applied, allowing the identification of a series of dual molecular diagnoses.

All the patients underwent a multi-step approach for the molecular diagnosis of HHL, summarized in Figure 1.

For all the patients enrolled in the study, the presence of mutations in the *GJB2*, *GJB6,* and *MT-RNR1* genes were previously excluded. In particular, the entire coding region of the *GJB2* gene was sequenced by the Sanger method (primers available upon request). DNA was analyzed on a 3500 Dx Genetic Analyzer (Thermo Fisher, Waltham, MA, USA), using ABI PRISM 3.1 Big Dye terminator chemistry (Thermo Fisher) according to the manufacturer’s instructions. Subsequently, *GJB6* deletions (i.e., D13S1830–D13S1854) were screened by multiplex PCR, as previously described [8], and the A1555G mtDNA mutation was tested by restriction fragment-length polymorphism (PCR-RFLP) analysis using BsmAI as restriction enzyme, followed by visualization on an agarose gel stained with Midori Green Advance (Nippon Genetics).

### 2.2. Multiplex Ligation Probe Amplification (MLPA)

MLPA analysis was performed for the identification of deletion/duplication in *STRC*, *CATSPER2,* and *OTOA* genes. The SALSA^®^ MLPA^®^ probe mixes P461-A1 DIS (MRC-Holland, Amsterdam, The Netherlands) were employed, according to the manufacturer’s instructions. Coffalyser. Net software was used for data analysis in combination with the lot-specific MLPA Coffalyser sheet. The following cutoff values for the dosage quotient (DQ) of the probes were used to interpret MLPA results: 0.80 < DQ < 1.20 (no deletion/duplication), DQ  =  0 (deletion), and 1.75 < DQ < 2.15 (duplication).

### 2.3. Whole-Exome Sequencing (WES)

WES was completed on an Illumina NextSeq 550 instrument (Illumina, San Diego, CA, USA) using the Twist Human Core Exome + Human RefSeq Panel kit (Twist Bioscience, South San Francisco, CA, USA), according to the manufacturer’s protocol.

Briefly, 50 ng of genomic DNA was enzymatically fragmented. After an end repair and dA-tailing reaction, each fragment was ligated to a universal adapter and then amplified using the Unique Dual Index primers (Twist Bioscience, South San Francisco, CA, USA). Afterward, the samples were hybridized with the Twist Human Core Exome and the Human RefSeq Panel, which allows covering 99% of the protein-coding genes. Finally, the hybridized fragments have been captured, amplified, and sequenced.

FASTQ files have been processed through a custom pipeline, called Germline-Pipeline, developed by enGenome s.r.l. (https://www.engenome.com/, accessed on 24 November 2021). This workflow has been designed for Illumina paired-end sequencing data and allows the identification of germline variants such as single-nucleotide variants (SNVs), short insertion/deletions (INDELs), exon-level copy number variations (CNVs) starting from sequence reads. The pipeline includes a series of steps, such as FASTQ trimming, FASTQ quality check, FASTQ mapping, mark of duplicates, base quality score recalibration, and variant calling.

A final VCF file containing SNV, INDELs, and CNV events has been generated.

The VCF files generated through the secondary analysis have been analyzed on Engenome Expert Variant Interpreter (eVai) software (evai.engenome.com), which allows variant annotation and interpretation. In particular, eVai combines artificial intelligence with the American College of Medical Genetics (ACMG) guidelines [9] to classify and prioritize every genomic variant, suggesting a list of possible related genetic diagnoses.

SNVs leading to synonymous amino acids substitutions not predicted as damaging, not affecting splicing, or highly conserved residues were excluded, as well as SNVs/INDELs with quality score (QUAL) < 20 and called in off-target regions.

A comparison between the identified genetic variants and data reported in NCBI dbSNP build153 (http://www.ncbi.nlm.nih.gov/SNP/, accessed on 24 November 2021) as well as in gnomAD (http://gnomad.broadinstitute.org/, accessed on 24 November 2021), led to the exclusion of those variants previously reported as polymorphism. In particular, a minor allele frequency (MAF) cutoff of 0.005 for recessive forms and 0.001 for the dominant ones was used.

The pathogenicity of known genetic variants was evaluated using ClinVar (http://www.ncbi.nlm.nih.gov/clinvar/, accessed on 24 November 2021), Deafness Variation Database (http://deafnessvariationdatabase.org/, accessed on 24 November 2021), and The Human Gene Mutation Database (http://www.hgmd.cf.ac.uk/ac/index.php, accessed on 24 November 2021).

Several in silico tools, such as PolyPhen-2 [10], SIFT [11], Pseudo Amino Acid Protein Intolerance Variant Predictor (for coding variants SNVs/INDELs) (PaPI score) [12], Deep Neural Network Variant Predictor (for coding/non-coding variants, SNVs) (DANN score) [13] and dbscSNV score [14] were used to evaluate the pathogenicity of novel variants.

Finally, on a patient-by-patient basis, variants were discussed in the context of phenotypic data at interdisciplinary meetings, and the most likely disease-causing SNVs/INDELs were analyzed by direct Sanger sequencing.

Sanger sequencing was also employed to perform the segregation analysis within the family.

## 3. Results

Among the 82 cases analyzed, a total of seven patients presented with a dual molecular diagnosis (8.5%). Based on both the genetic and clinical findings, patients have been classified into two main categories: (1) those with distinct phenotypes, i.e., HL plus other clinical features due to mutations in different genes, (2) patients with overlapping phenotypes, i.e., HL due to the contribution of two distinct loci.

In particular, five families belong to the first class of patients (i.e., Family 1, 2, 3, 4, 5), while the remaining two belong to the second one (i.e., Family 6, 7).

### 3.1. Family 1. Hearing Loss and Myopathy

**Figure 2:**
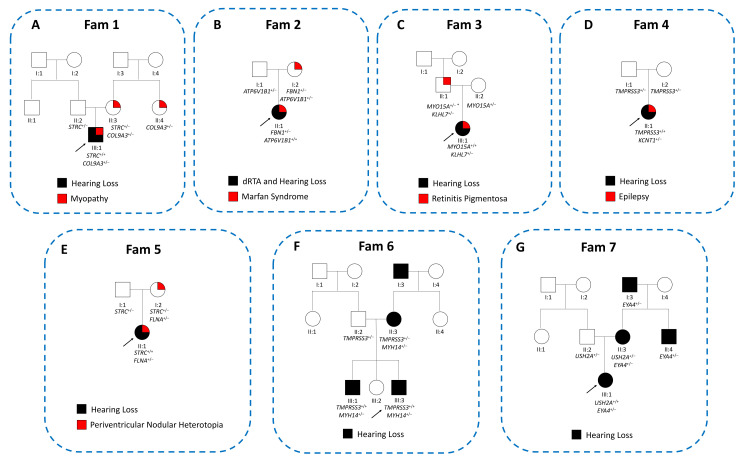
Pedigrees, clinical and genetic data of the families carrying a dual molecular diagnosis. The figure illustrates all the pedigrees of the HL families with a dual molecular diagnosis, with the indication of the main clinical features and the genes involved in the phenotype (i.e., Families 1–7, which correspond to Figure 1, (**A**–**G**). dRTA = distal renal tubular acidosis. * Individual carrier of two *in cis* missense variants in the *MYO15A* gene.

Fam 1 (Figure 2A), an Italian non-consanguineous family, came to genetic counseling for the presence of an apparent NSHL in the proband, an 11-year-old boy.

The audiometric evaluation revealed a mild-moderate HL across all frequencies and normal hearing in both parents (Supplementary Table S1). Family history highlighted the presence of myopathy of unknown origin in the mother (confirmed by muscle biopsy), presenting with muscular weakness of the left arm, myotonic firing, and progressive involvement of the inferior part of the body. The maternal aunt reported a similar condition, and the mother outlined the presence of muscle and joint pain also in the proband.

The family trios underwent our multi-step approach for the molecular diagnosis of HL. The analysis of CNVs in *STRC*, *CATSPER2*, and *OTOA* genes through MLPA revealed the presence of a homozygous *STRC* (NM_153700.2) deletion in the proband (Supplementary Figure S1A, Table 1), inherited from the parents (both heterozygous).

As the second step, the family trio has been sequenced by WES to investigate the molecular basis of the familiar myopathy.

Data analysis revealed the presence of a likely pathogenic missense variant in *COL9A3* (NM_001853.4) (Supplementary Figure S1B, Table 1), a gene causative of epiphyseal dysplasia, type 3, with or without myopathy (MIM#: 600969, ORPHA: 166002) with an autosomal dominant pattern of inheritance. The c.1378G > A, p.(Gly460Ser) variant locates in the exon 27 and affects a highly conserved residue. It is predicted as damaging by the *in silico* tools used during data analysis and was found at the heterozygous state both in the mother and the proband, likely explaining the clinically diagnosed myopathy. Segregation analysis in the maternal aunt showed the presence of the variant in her too.

### 3.2. Family 2. Hearing Loss, Distal Renal Tubular Acidosis (dRTA), and Marfan Syndrome

Fam 2 (Figure 2B), an Italian non-consanguineous family, came to the geneticist’s attention for a suspect of familiar Marfan syndrome.

In particular, both the proband, a 10-year-old girl, and the mother showed a series of clinical features attributable to Marfan syndrome (e.g., long, narrow face, micrognathia, disproportionate tall stature, arachnodactyly, etc.). In addition, an accurate clinical evaluation that included hematological and biochemical tests revealed the presence of metabolic acidosis (pH 7.25), hyperchloremia, and hypokalemia in the proband, suggesting a clinical diagnosis of distal renal tubular acidosis (dRTA). Furthermore, the proband displayed a congenital progressive asymmetric HL (moderate to severe in the right ear and severe to profound in the left one) (Supplementary Table S1).

The family trio was analyzed through WES, which highlighted the presence of: (1) a nonsense mutation already described as causative of Marfan syndrome (MIM#: 154700, ORPHA: 558) [15] in *FBN1* (NM_000138.5) (c.4930C > T, p.(Arg1644*)), at the heterozygous state both in the proband and the mother, and (2) two compound heterozygous mutations already described as causative of dRTA and HL (MIM#: 267300, ORPHA: 18) [16,17], the c.242T > C, p.(Leu81Pro) and the c.687 + 1G > A, in the *ATP6V1B1* gene (NM_001692.4) in the proband (Supplementary Figure S1C,D, Table 1).

### 3.3. Family 3. Hearing Loss and Retinitis Pigmentosa

Fam 3 (Figure 2C) came to genetic counseling for a problem of hearing loss and retinitis pigmentosa (RP) in the proband, a 30-year-old Italian woman with severe congenital deafness (Supplementary Table S1) and the onset of RP at the age of 25 years. The first diagnostic hypothesis was that of Usher syndrome; however, the collection of the family history revealed the presence of RP also in the father and paternal grandmother (onset at approximately 20 years in both), suggesting an autosomal dominant inheritance of this trait.

WES of the family trio revealed the presence in the proband of three missense variants in the *MYO15A* gene (NM_016239.3) (Supplementary Figure S1E, Table 1), which is known to be associated with sensorineural non-syndromic autosomal recessive deafness (DNF3B, MIM#: 600613). The first one is the c.3742C > T, p.(Arg1248Trp) mutation, inherited from the mother and already reported as pathogenic of autosomal recessive NSHL [18]. The other two variants, namely c.6370C > T, p.(Arg2124Trp) and c.5473G > A p.(Val1825Met), are both inherited from the father. None of them is already described as clearly pathogenic; however, they affect highly conserved residues, are rare (MAF: 0.004% and 0.012%, respectively), and are predicted as damaging by all the in silico tools used during data analysis. Furthermore, a distinct mutation affecting one of these residues (p.(Arg2124Gln)) was identified as the cause of NSHL in a family described in the literature [19].

Additionally, WES led to the identification in the proband and in her father of a known heterozygous pathogenic mutation in *KLHL7* (NM_001031710.2) (c.458C > T p.(Ala153Val)) (Supplementary Figure S1F, Table 1) already described in a family with late-onset autosomal dominant RP [20] (MIM#: 612943).

### 3.4. Family 4 Hearing Loss and Epilepsy

The proband of Fam 4 (Figure 2D) is a 27-year-old Italian woman, a unique child of non-consanguineous healthy parents, with unremarkable family history. At 18 months of age, she was diagnosed with a congenital profound sensorineural hearing loss (Supplementary Table S1). Sequencing analysis of the gene *GJB2* (coding for connexin-26) and analysis for the deletion involving delGJB6-DS13S1830 of GJB6, coding for connexin 30, were negative. Despite implantation of the bilateral external prosthesis, her developmental milestones were characterized by a cognitive delay with predominant language impairment. When she was nine years old, she presented with seizures with sudden arousal, eye opening, diffuse rigidity, and limbs jerks arising from sleep. Based on EEG pattern and video-polysomnography (which documented up to 40 motor events/night mainly characterized by asymmetric tonic posturing), a diagnosis of sleep-related hypermotor epilepsy was made. In the hypothesis of a syndrome associating HL and epileptic encephalopathy, arrayCGH analysis was performed without identifying any copy number variation. WES was then performed on the family trio. Data analysis revealed the presence in the proband of a de novo heterozygous mutation in *KCNT1* (NM_020822.3) (c.2800G > A, p.(Ala934Thr)) [21] (Supplementary Figure S1G, Table 1**)**. *KCNT1* mutations are a known cause of dominant epileptic encephalopathy (MIM#: 614959), and the same mutation was previously described as pathogenic [22]. Segregation analysis by Sanger sequencing confirmed that the mutation occurred as de novo in the proband, being absent in the patient’s parents.

Additionally, two likely pathogenic variants in *TMPRSS3* (NM_024022.2), a gene known for being causative of autosomal recessive deafness (MIM#: 601072, ORPHA: 90636), were identified in the proband. In particular, the proband carried a pathogenic frameshift deletion (c.208delC, p.(His70Thrfs*19)) inherited from her father and a pathogenic missense mutation (c.1211C > T, p.(Pro404Leu)) on the maternal allele (Supplementary Figure S1H, Table 1); both the mutations were previously described as causative of childhood NSHL [23,24].

### 3.5. Family 6. Familiar Hearing Loss

Fam 6 (Figure 2F) is an Italian non-consanguineous family characterized by a likely dominant NSHL.

A careful anamnesis revealed the presence of NSHL in the proband, in the older brother, and in the mother.

In particular, the proband, a nine years old boy, developed moderate bilateral symmetric HL at the age of three that worsened during the years, becoming severe to profound at the medium and high frequencies. The brother (22 years old) showed a similar audio profile, despite being diagnosed later (concomitantly with the proband). The mother (46 years old) displayed a milder phenotype, with a moderate HL at the medium and high frequencies, diagnosed at the age of 25 (Supplementary Table S1). Moreover, the maternal grandfather reported hearing loss, too, developed during the third decade of life (audiometric test not available).

Further clinical assessments did not reveal any other relevant features.

The proband was first screened for mutations in connexin genes, for the mitochondrial mutation A1555G, and for the presence of CNV in *STRC*, *CATSPER2*, and *OTOA* genes, with negative results.

WES was then performed on the family trio. Data analysis revealed the presence in the proband of two likely pathogenic variants in the *TMPRSS3* gene, known for being causative of autosomal recessive deafness (MIM#: 601072, ORPHA: 90636), together with a likely pathogenic variant in *MYH14*, a gene described for causing autosomal dominant HL (MIM#: 600652, ORPHA: 90635) (Supplementary Figure S1I,J Table S1).

As regards *TMPRSS3* (NM_024022.2), the c.413C > A, p.(Ala138Glu) mutation in exon 5 and the c.977C > T, p.(Pro326Leu) variant in exon 10 have been identified. The first mutation, inherited from the father, is already described as causative of childhood NSHL by both the HGMD Professional database [25] and Deafness Variation Database. The second variant, inherited from the mother, is not reported in any public database; it affects a highly conserved residue and is predicted as damaging by all the *in silico* tools used during the tertiary analysis.

Concerning *MYH14* (NM_024729.3), the c.160C > A, p.(Arg54Ser) variant in exon 2 was detected at the heterozygous state in both the proband and the affected mother. The variant affects a highly conserved residue, is predicted as damaging, and is not described in any public database.

Sanger sequencing confirmed the presence of all the variants also in the affected brother.

Furthermore, two additional families, Family 5 (Figure 2E, belonging to the first group of patients) and Family 7 (Figure 2G, belonging to the second group of patients), have already been described in our previous work [5].

Briefly, Family 5 came to genetic counseling for a mild-moderate HL and periventricular nodular heterotopia, the latter being present both in the proband and in the mother. Our multi-step approach led to the identification of a homozygous deletion in the STRC gene in the proband, explaining the HL phenotype, while WES pointed out a nonsense variant, i.e., c.1159C > T, p.(Gln387*), in *FLNA* gene (NM_001456.3) (Table 1), a gene causative of periventricular nodular heterotopia in an X-linked dominant fashion (MIM#: 300049, ORPHA: 98892) [26].

The second family, Family 7, was characterized by a likely dominant NSHL, with the presence of HL in the proband, in the mother, in the maternal uncle, and maternal grandfather.

WES data analysis revealed the presence of: (1) a nonsense variant segregating in all the affected family members in *EYA4* (NM_004100.5) (c.714C > A, p.(Tyr238*)), a gene known for being causative of autosomal dominant NSHL (MIM#: 601316, OPRHA: 90635), and (2) two pathogenic mutations in *USH2A* gene (NM_206933.2) at the compound heterozygous state (i.e., c.11864G > A, p.(Trp3955*) and c.2276G > T, p.(Cys759Phe)), which is known for being causative of Usher syndrome type 2A (MIM#: 276901, ORPHA:886) in the proband, a girl of three years old (Table 1).

## 4. Discussion

The presence of dual molecular diagnoses is becoming a more and more frequent finding in all genetic conditions. In fact, several reports demonstrated that this scenario occurs in a relatively large percentage of cases, ranging from 4.6% to 8.9% of the patients who received a molecular diagnosis [27,28,29]. Moreover, some previous works suggest that patients with multiple diagnoses might be under-recognized, hypothesizing that the frequency of multiple diagnoses could be even higher [28].

Data here presented confirmed the presence of dual molecular diagnoses also in HL patients suggesting they can occur in a large percentage of cases. In particular, among the 82 families recruited during the last year, seven display a dual diagnosis, for an overall percentage of 8.5% of the total cases and 17.9% of all the patients who received a molecular diagnosis. Of course, we should take into account that these results have been obtained from a small sample size, which might be enriched by complex scenarios. All the cases have been selected from a larger group of patients enrolled in our institute, excluding *GJB2*-positive patients, and dual diagnoses might be more frequent in individuals displaying a variety of clinical features. In this light, to clearly define the contribution of dual diagnosis in the etiopathogenesis of HHL, it is necessary to expand the number of analyzed samples. However, these results point out the complexity of the molecular basis of HHL, highlighting that the identification of a single molecular diagnosis might not be conclusive for all the cases, thus requiring a deeper data evaluation.

Based on this evidence, it appears clear that even in the genomic era, it is still fundamental to have extensive knowledge of the investigated phenotype in order to critically interpret the sequencing findings. For this reason, sequencing data analysis should be performed by a multidisciplinary team of geneticists, clinicians, radiologists, and otolaryngologists that altogether cooperate with the final aim of defining the correct molecular diagnosis.

The examples mentioned above suggest that possibility of a dual molecular diagnosis in HHL families should be taken into consideration in the following cases:

(1) in the presence of patients with clinical features that do not fit into a known model/syndrome. In this case, the possible scenarios include the identification of a novel syndrome, with a new gene involved, or, as shown in patients of Fam 1, 2, 3, 4, 5, the presence of dual independent genetic causes;
(2) in the presence of families with a high intra-familial phenotypic variability. As shown for the probands of Family 6 and 7, the differences in the degree or onset of HL might be the consequence of concomitant genetic causes that modulate and worsen the clinical manifestation.

The knowledge of the genetic background responsible for HL is extremely valuable in many ways: it helps in elucidating the biology of the hearing system, and, more importantly, has relevant practical outcomes, influencing the clinical management of the patients, providing recurrence risk estimation and paving the way for the development of future personalized therapeutic approaches.

Overall, the examples described here point out the emerging role of dual molecular diagnosis in patients affected by both SHL and NSHL, suggesting that alternative inheritance scenarios should be taken into account, especially for those cases that depart from the expected clinical manifestation. Moreover, given the high proportion of dual diagnosis here detected, the recommended approach for the study of the genetics of HL should include NGS technologies, which are able to provide an unbiased screen of our genome.

We conclude that, once the most common forms of SHL are excluded, the frequency of dual molecular diagnosis can be as high as 10% in the remaining cases. Furthermore, dual molecular diagnosis can explain a small but relevant proportion of families with clinically heterogeneous NSHL thus should be carefully considered for the early diagnosis and the clinical management of the patients.

## Figures and Tables

**Table 1:** List of dual molecular diagnoses identified by WES and MLPA. The table displays the main clinical features and the variants detected in the probands with a dual molecular diagnosis. Hom = homozygous; Het = heterozygous; NA = not available.The * symbol indicates a stop codon in the protein, according to the HGVS nomenclature guidelines.

		Gene 1					Gene 2				
Family ID	Clinical Phenotype	Gene	cDNA Change	Genotype	Protein Change	Inheritance	Gene	cDNA Change	Genotype	Protein Change	Inheritance
1	Hearing loss and myopathy	*STRC* (NM_153700.2)	gene deletion	Hom	NA	maternal + paternal	*COL9A3* (NM_001853.4)	c.1378G > A	Het	p.(Gly460Ser)	maternal
2	Hearing loss, distal renal tubular acidosis (dRTA) and Marfan syndrome	*ATP6V1B1* (NM_001692.4)	c.242T > C	Het	p.(Leu81Pro)	maternal	*FBN1* (NM_000138.5)	c.4930C > T	Het	p.(Arg1644*)	maternal
			c.687 + 1G > A	Het	NA	paternal					
3	Hearing loss and retinitis pigmentosa	*MYO15A* (NM_016239.3)	c.3742C > T	Het	p.(Arg1248Trp)	maternal	*KLHL7* (NM_001031710.2)	c.458C > T	Het	p.(Ala153Val)	paternal
			c.6370C > T	Het (*in cis* with c.5473G > A)	p.(Arg2124Trp)	paternal					
			c.5473G > A	Het (*in cis* with c.6370C > T)	p.(Val1825Met)	paternal					
4	Hearing loss and epilepsy	*TMPRSS3* (NM_024022.2)	c.1211C > T	Het	p.(Pro404Leu)	maternal	*KCNT1* (NM_020822.3)	c.2800G > A	Het	p.(Ala934Thr)	de novo
			c.208delC	Het	p.(His70Thrfs*19)	paternal					
5	Hearing loss and periventricula nodular heterotopia	*STRC* (NM_153700.2)	gene deletion	Hom	NA	maternal + paternal	*FLNA* (NM_001456.3)	c.1159C> T	Het	p.(Gln387*)	maternal
6	Hearing loss	*TMPRSS3* (NM_024022.2)	c.413C > A	Het	p.(Ala138Glu)	paternal	*MYH14* (NM_024729.3)	c.160C > A	Het	p.(Arg54Ser)	maternal
			c.977C > T	Het	p.(Pro326Leu)	maternal					
7	Hearing loss	*EYA4* (NM_004100.5)	c.714C > A	Het	p.(Tyr238*)	maternal	*USH2A* (NM_206933.2)	c.2276G > T	Het	p.(Cys759Phe)	paternal
								c.11864G > A	Het	p.(Trp3955*)	maternal

## Data Availability

The data presented in this study are available upon request from the corresponding author. The data are not publicly available due to privacy restrictions.

## Supplementary Materials

The following are available online at https://www.mdpi.com/article/10.3390/biomedicines10010012/s1, Figure S1: Genetic findings of the families carrying a dual molecular diagnosis and protein sequence alignment across species, Table S1: Audiometric features of all the patients and relatives enrolled in the study.
