# Supplementary material for: There Is More Than Meets the Eye: Identification of Dual Molecular Diagnosis in Patients Affected by Hearing Loss
Source: Biomedicines. 2021 Dec 22;10(1):12. doi: 10.3390/biomedicines10010012 (PMC8773038; doi:10.3390/biomedicines10010012)
Supplement: Supplementary file 1 [file Figure_S1.pdf › Figure_S1.pdf]

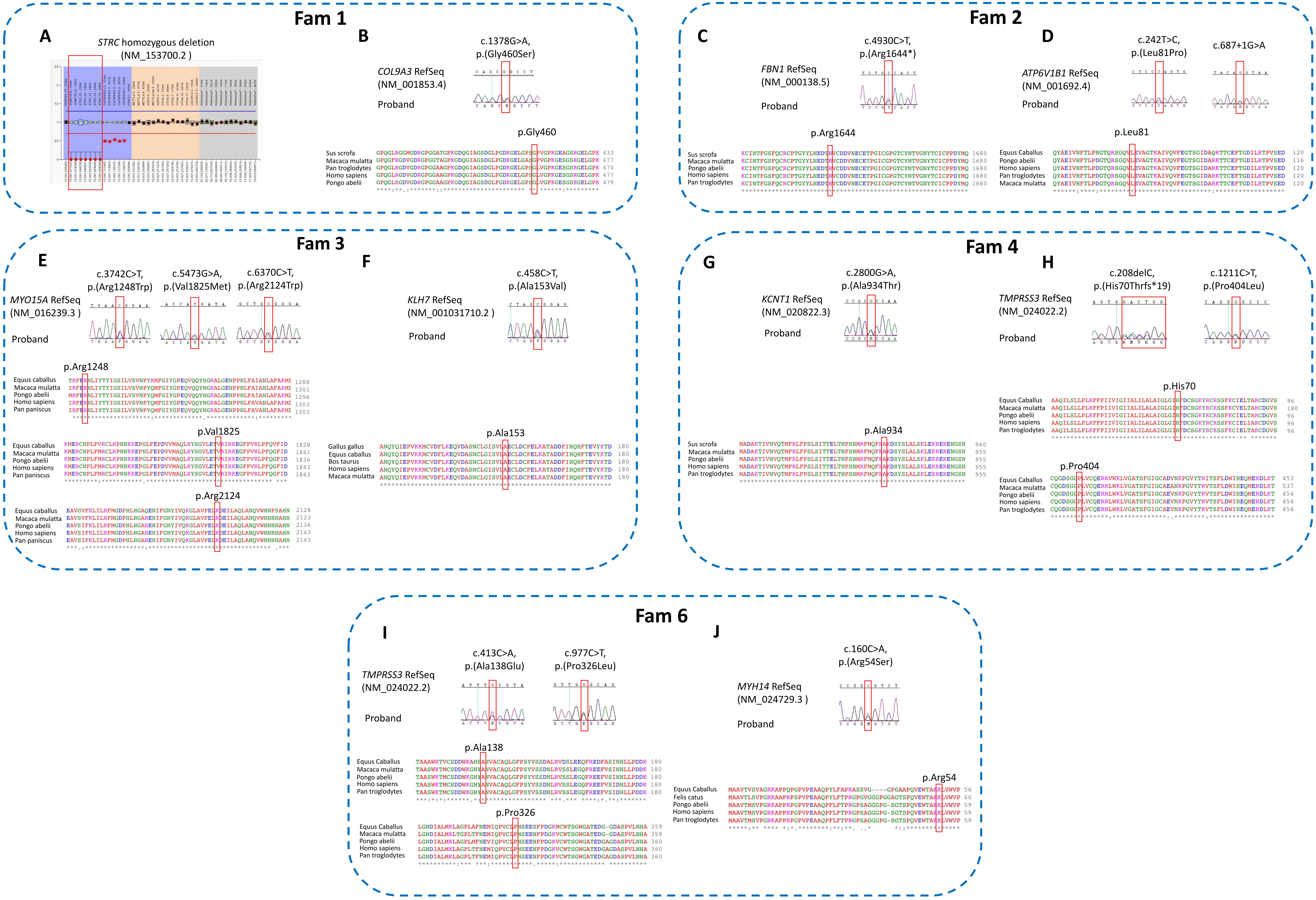
Fam 1
A
STRC homozygous deletion
(NM_153700.2)
B
c.1378G>A,
p.(Gly460Ser)
COL9A3 RefSeq
(NM_001853.4)
Proband
p.Gly460
Sus scrofa
Macaca mulatta
Pan troglodytes
Homo sapiens
Pongo abelii
Fam 2
C
c.4930C>T,
p.(Arg1644*)
FBN1 RefSeq
(NM_000138.5)
Proband
p.Arg1644
Sus scrofa
Macaca mulatta
Pongo abelii
Homo sapiens
Pan troglodytes
D
c.242T>C,
p.(Leu81Pro)
c.687+1G>A
ATP6V1B1 RefSeq
(NM_001692.4)
Proband
p.Leu81
Equus Caballus
Pongo abelii
Homo sapiens
Pan troglodytes
Macaca mulatta
Fam 3
E
c.3742C>T,
p.(Arg1248Trp)
c.5473G>A,
p.(Val1825Met)
c.6370C>T,
p.(Arg2124Trp)
MYO15A RefSeq
(NM_016239.3)
Proband
p.Arg1248
Equus caballus
Macaca mulatta
Pongo abelii
Homo sapiens
Pan paniscus
p.Val1825
p.Arg2124
F
c.458C>T,
p.(Ala153Val)
KLH7 RefSeq
(NM_001031710.2)
Proband
p.Ala153
Gallus gallus
Equus caballus
Bos taurus
Homo sapiens
Macaca mulatta
Fam 4
G
c.2800G>A,
p.(Ala934Thr)
KCNT1 RefSeq
(NM_020822.3)
Proband
p.Ala934
Sus scrofa
Macaca mulatta
Pongo abelii
Homo sapiens
Pan troglodytes
H
c.208delC,
p.(His70Thrfs*19)
c.1211C>T,
p.(Pro404Leu)
TMPRSS3 RefSeq
(NM_024022.2)
Proband
p.His70
Equus Caballus
Macaca mulatta
Pongo abelii
Homo sapiens
Pan troglodytes
p.Pro404
Fam 6
I
c.413C>A,
p.(Ala138Glu)
c.977C>T,
p.(Pro326Leu)
TMPRSS3 RefSeq
(NM_024022.2)
Proband
p.Ala138
Equus Caballus
Macaca mulatta
Pongo abelii
Homo sapiens
Pan troglodytes
p.Pro326
J
c.160C>A,
p.(Arg54Ser)
MYH14 RefSeq
(NM_024729.3)
Proband
p.Arg54
Equus Caballus
Felis catus
Pongo abelii
Homo sapiens
Pan troglodytes
